# Global Analyses and Latest Research Hot Spots of Adipose-Derived Stem Cells in Fat Grafting: A Bibliometric and Visualized Review

**DOI:** 10.1007/s00266-022-03201-1

**Published:** 2022-12-02

**Authors:** Tian-Hao Li, Zi-Ming Li, Xiao-Han Qin, Nan-Ze Yu, Jiu-Zuo Huang, Xiao Long

**Affiliations:** 1 Department of Plastic and Reconstructive Surgery, Peking Union Medical College Hospital, Chinese Academy of Medical Sciences, Peking Union Medical College, Beijing, China; 2 Department of Cardiology, Peking Union Medical College Hospital, Chinese Academy of Medical Sciences, Peking Union Medical College, Beijing, China

**Keywords:** Bibliometric, Adipose-derived stem cells, Fat grafting, Lipoinjection

## Abstract

**Background:**

Fat grafting is one of the most effective treatments for soft tissue restoration and augmentation. Adipose-derived stem cells (ASCs) supplementation is one of the foremost concerns to improve its efficiency. There have been several studies aiming at adipose-derived mesenchymal stem cells in fat grafting, but no relevant bibliometric research has conducted.

**Methods:**

Articles about fat grafting and ASCs were retrieved in Web of Science Core Collection (WoSCC). Using VOSviewer 1.6.10.0 (Leiden University, the Netherlands) and CiteSpace 6.1.R2 (Drexel University, USA), the information of national distribution, institutions, journals, authors and keywords were evaluated and calculated.

**Results:**

A total of 1166 papers in the field of ASCs in fat grafting were retrieved from 2002 to 2021. The USA produced the most articles, and the top 2 productive institutions were all from the USA. Researchers and institutions conducting ASCs in fat grafting research have shown a widespread and close connection. Plastic and Reconstructive Surgery published the most article on ASCs in fat grafting, and professor Rubin Peter is the most productive author. The top 10 references with the highest LCS mainly focused on applying ASCs to assist fat transplantation in plastic surgery. The most cited keywords formed 4 clusters, and “mesenchymal stem,” “mesenchymal stromal cell,” “stromal vascular fraction” and “long term” were the most recently trending keywords.

**Conclusions:**

This article provides a summary of the current research status focusing on fat grafting and ASCs. More efforts will be made to promote the application of ASCs in fat grafting.

**Level of Evidence V:**

This journal requires that authors assign a level of evidence to each article. For a full description of these Evidence-Based Medicine ratings, please refer to the Table of Contents or the online Instructions to Authors www.springer.com/00266.

**Supplementary Information:**

The online version contains supplementary material available at 10.1007/s00266-022-03201-1.

## Introduction

Autologous fat grafting is one of the most effective and safest technique in reconstructive surgery to promote functional and esthetic form [1]. It has several advantages such as accessibility to adipose tissue, inexpensive, non-immunogenicity and biocompatibility. However, grafted fat resorption is still a major issue to be solved to improve graft retention [2]. Adipose tissue which is non-vascularized could not bear ischemic or oxidative stress after grafted and could lead to uncertain survival of adipocytes. Using adipose tissue with a stromal vascular fraction (SVF), a heterogeneous cell mixture including adipose-derived stem cells (ASCs), may improve the long-term stability of fat grafting [3, 4]. Similar to mesenchymal stromal cells from bone marrow, the ASCs could differentiate into multiple cell lines such as adipocytes, myocytes, osteoblasts and chondrocytes [1]. It is important to summarize the research status in this field and further study is needed to facilitate the application of ASCs in fat grafting.

Bibliometric reviews analyze the national and institutional characteristics, quantity change, journals, authors and citation patterns of the publications collected from the WoS Core Collection database (WoSCC) [5]. Bibliometrics has been conducted in several diseases and medical technologies, such as pneumonia, arthritis, hypertension and HIV [6–9]. However, there is no bibliometric study focusing on ASCs in fat grafting, which is a valuable research topic to improve clinical treatment. So, we conducted a bibliometric review on ASCs combined with fat grafting and analyzed the number and information of the publications including publication years, document types, research areas, citations, journals, authors, countries/regions, languages, affiliations and keywords, aiming to depict a comprehensive research status and point out some future perspectives.

## Materials and Methods

### Search Strategies and Data Acquisition

The data in this study was retrieved from the WoSCC, one of the most comprehensive databases, in September 7, 2022. Referring to our pre-study, the search strategy was set as “(TS = (Adipose derived Stem Cells) OR TS = (Adipose-Derived Mesenchymal Stem Cells) OR TS = (Adipose Derived Mesenchymal Stem Cells) OR TS = (Mesenchymal Stem Cells, Adipose-Derived) OR TS = (Adipose Derived Mesenchymal Stromal Cells) OR TS = (Adipose-Derived Mesenchymal Stromal Cells) OR TS = (Adipose Tissue Derived Mesenchymal Stromal Cells) OR TS = (mesenchymal stem cells)) AND (TS = (fat grafting) OR TS = (lipografts) OR TS = (lipoinjection) OR TS = (lipotransfer) OR TS = (fat transfer) OR TS = (fat transplant) OR TS = (lipostructure lipofilling))” within the publication year span from 2002-01-01 to 2021-12-31. The total of 1235 publications were retrieved and 1216 articles were acquired after removing non-English articles. After excluding meeting abstract, case report, editorial material, letter, correction and other document types, 1166 publications of articles or reviews were included. Data including the articles and citations, H-index, keywords, publication years, journals, authors, countries/regions and affiliations were retrieved from WoSCC.

### Bibliometric Analysis

Using bibliographic VOSviewer 1.6.10.0 (Leiden University, the Netherlands), CiteSpace 6.1.R2 (Drexel University, USA), HiteSpace Pro 2.0 (Thomson Reuters, Canada), the web tool (https://www.bioinformatics.com.cn/) and Excel 2016 software, statistical analyses were performed based on the retrieved data. The bibliographic data was exported as a txt file from WoSCC and then imported into VOSviewer and CiteSpace software to generate visualization maps. The productive capacity was quantified by the number of publications (Np), and the article impact was evaluated by the number of citations (Nc). H-index, which combined productive capacity and impact by excavating the threshold connecting Np and Nc, was used to assess the contribution of researchers and forecast future research findings [10, 11].

VOSviewer software was applied to generate bibliometric network. Using this, we performed the co-citation and co-occurrence analysis in our study. The size of the nodes indicated the number of publications. The line thickness indicated the strength of the association. Different clusters were shown with different colors [12]. CiteSpace software was commonly used in information visualization analyses [13]. It was applied to mark keywords, perform cluster analyses and identify knowledge development timeline and emerging trends. HistCite software was mostly applied to translate citation relationships into paths, which could provide more explanatory visual analysis and developmental traits of the researches [14].

## Results

### An Overall Characteristic of the Publications

There were a total of 1166 publications matching the search strategy, including 972 articles and 194 reviews, and were then involved in further bibliometric analyses. For all publications, there were a total of 42816 citations, of which 36393 without self-citations. The average citations per document were 36.72, and the H-index of all publications was 93.

### Temporal Trend of Annual Publication Quantity

**Fig. 1:**
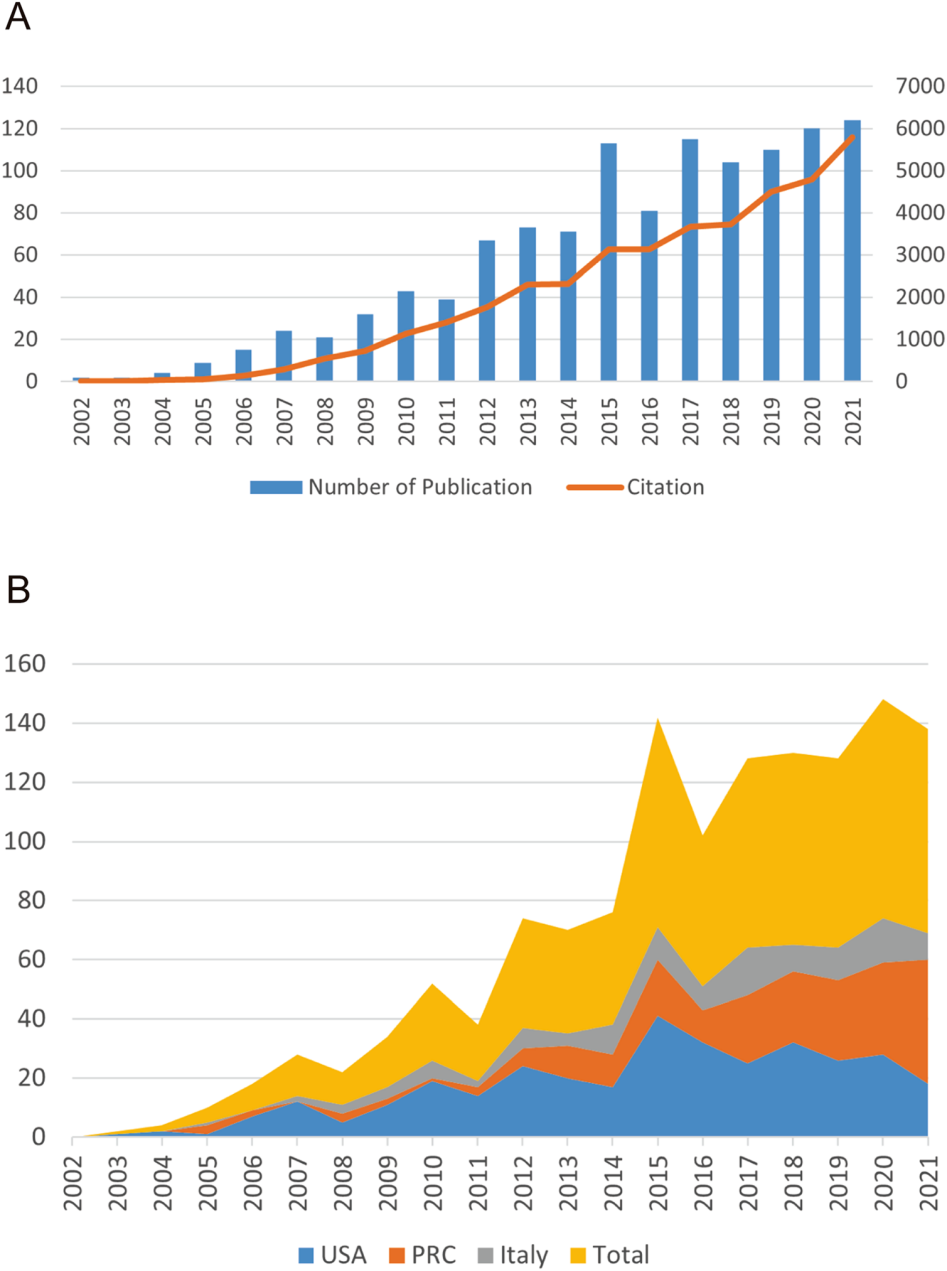
**A** Number of publications by year over the past 10 years. **B** Annual publications and cumulative citations from 2002 to 2021

The annual Np associated with ASCs and fat grafting is shown in Fig. 1. The top 3 most highly productive countries were the USA (Np = 335), China (Np = 219) and Italy (Np = 118) with the proportion of 57.6% of the 1166 publications. The trend of annual publication quantity and the polynomial-fitting curve of annual citation quantity are shown in Fig. 1. The number of annual publishing articles increased from 2 in 2002 to a peak of 124 in 2021. There was a significant correlation between annual Nc and the publication year with the correlation coefficient *R*^2^ reaching 0.9298. These showed that ASCs in fat grafting was a research hot spot and researchers have been keeping on investigating on this issue (Table 1).

**Table 1.**
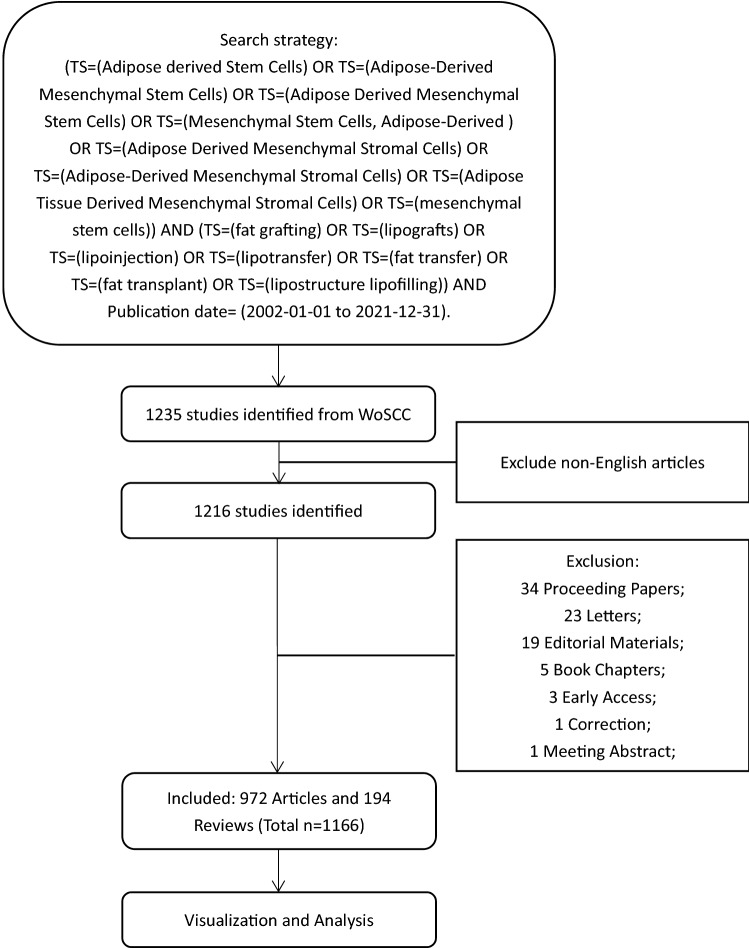
Flowchart of the screening process.

### The National/Regional Distribution of Global Publications

**Fig. 2:**
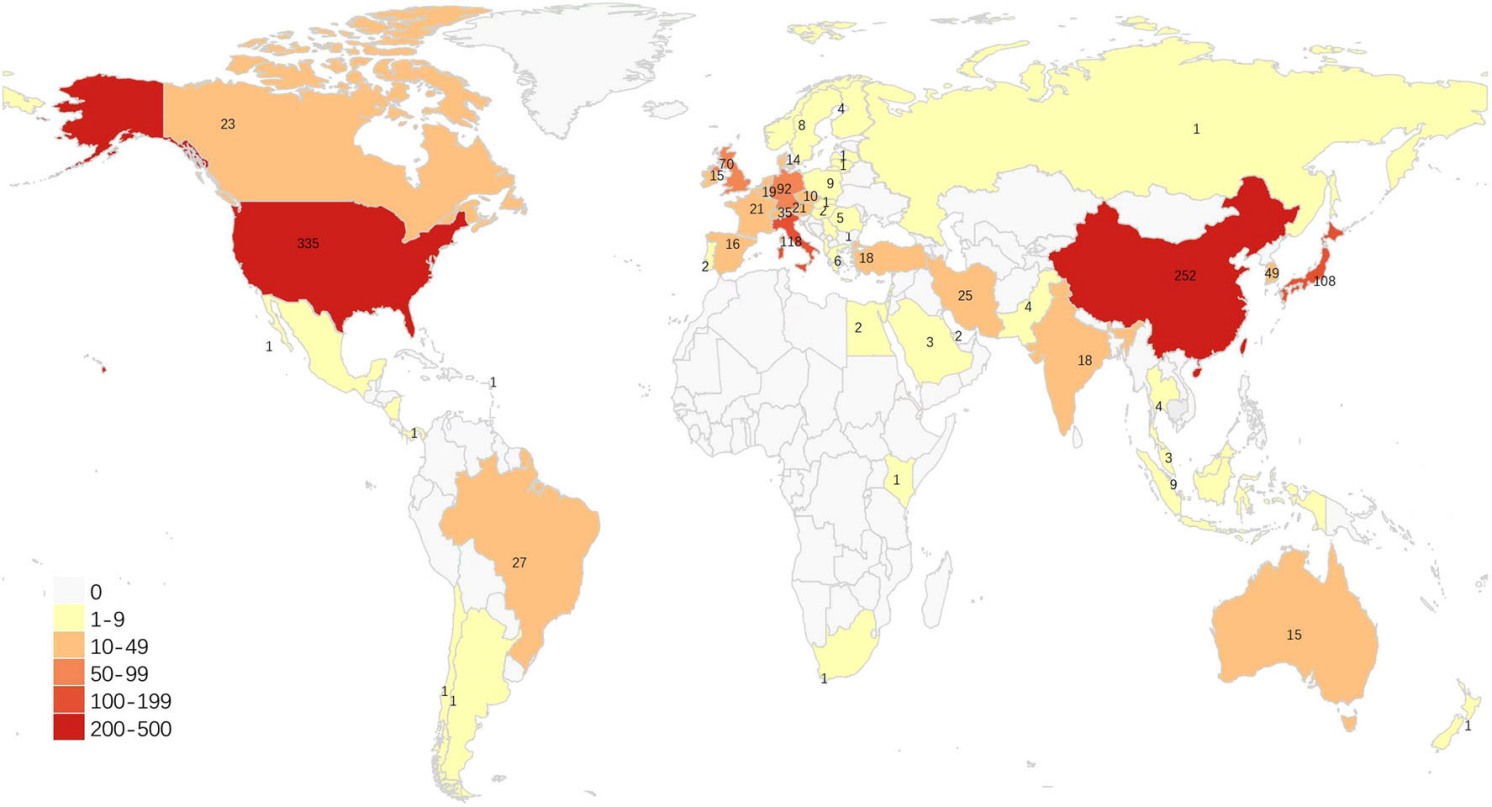
Graphical distribution of publications of adipose-derived stem cells in fat grafting, 2002-2021.

**Fig. 3:**
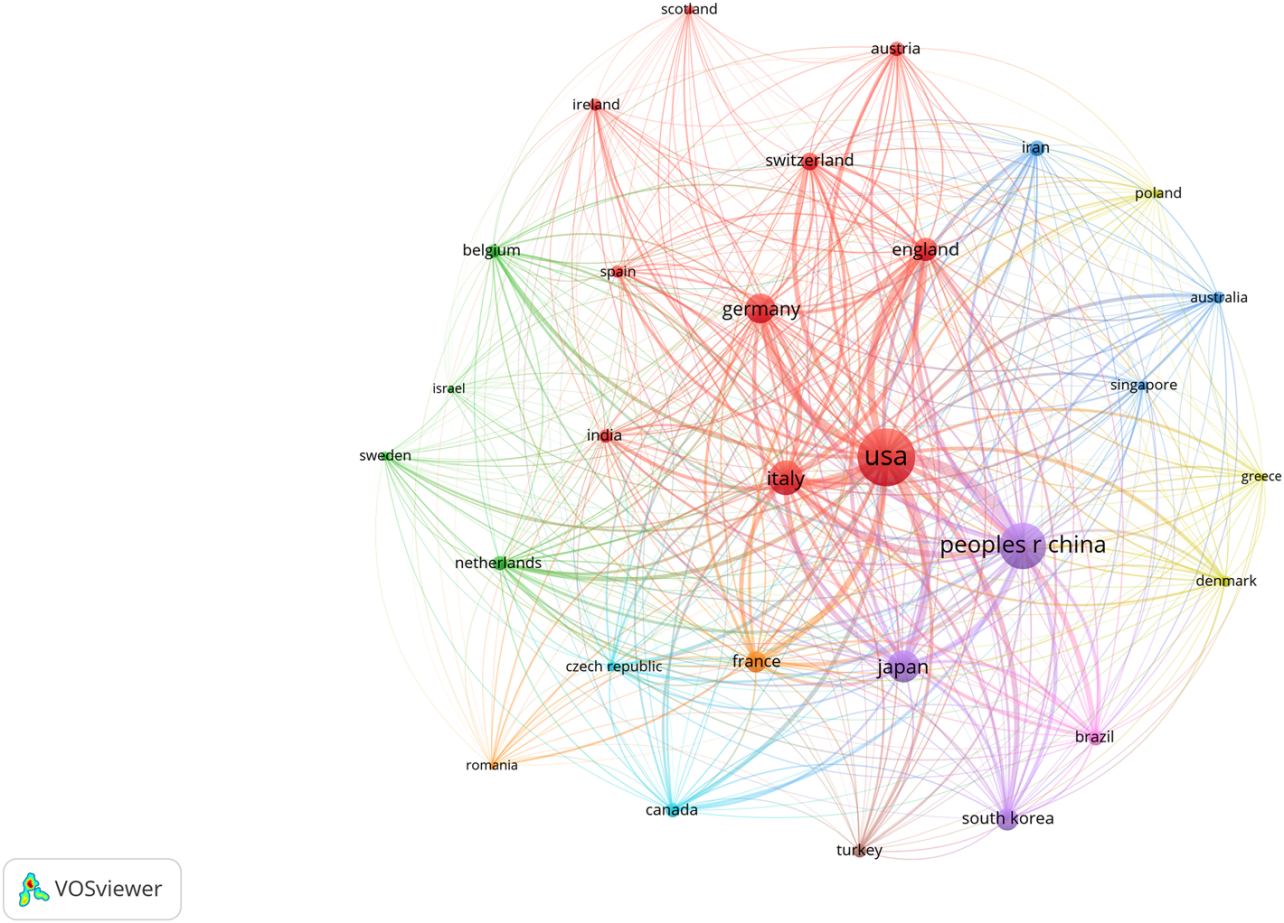
Network map for global country distribution analysis of adipose-derived stem cells in fat grafting research, from 2012 to 2021.

The distribution of countries/regions of the global publications related to ASCs and fat grafting is shown in Fig. 2. The ranking of the top 10 most productive countries/regions is shown in Table 2. We used VOSviewer software to perform the countries/regions distribution analysis with the minimum number of documents of one country set as 5. A total of 30 countries met the standard and were visualized with a network diagram and a density map as shown in Fig. 3 and Fig. S1. USA owned the largest number of published articles, and China and Italy were in second and third place, respectively. In addition, USA had the highest H-index, followed by Italy and Japan tied for second place.

**Table 2.** Top 10 countries/regions according to publications.

Rank	Country/region	Np	% of (1166)	Nc	H-index
1	USA	335	28.73%	15707	63
2	Peoples R China	219	18.78%	4310	32
3	Italy	118	10.12%	4836	40
4	Japan	108	9.26%	6897	40
5	Germany	92	7.89%	2893	28
6	England	58	4.97%	1723	22
7	South Korea	49	4.20%	1356	19
8	France	47	4.31%	1553	23
9	Switzerland	35	3.00%	902	15
10	Taiwan	33	2.83%	705	17

### Distribution of Affiliations

The top 10 affiliations focusing on ASCs and fat grafting are listed in Table 3. The top 3 affiliations owing largest number of publications were mostly from the USA, that were University of California System (Np = 49) and Pennsylvania Commonwealth System of Higher Education PCSHE (Np = 37). The Pennsylvania Commonwealth System of Higher Education PCSHE had the highest H-index of 25, followed by the University of Pittsburgh having the H-index of 24.

**Table 3.** Top 10 affiliations according to publications.

Rank	Affiliations	Country	Np	% of (1166)	Nc	H-index
1	University of California System	USA	49	4.20%	2014	19
2	Pennsylvania Commonwealth System of Higher Education PCSHE	USA	37	3.17%	2182	25
3	Southern Medical University China	China	37	3.17%	654	15
4	University of Pittsburgh	USA	35	3.00%	1685	24
5	Institute National DE LA Sante ET DE LA Recherche Medical Inserm	France	25	2.14%	729	16
6	Stanford University	USA	24	2.06%	930	15
7	University of Tokyo	Japan	23	1.97%	3298	19
8	University of London	England	21	1.80%	401	10
9	University of Rome Tor Vergata	Italy	21	1.80%	1210	18
10	University of Toronto	USA	21	1.80%	1288	16

**Fig. 4:**
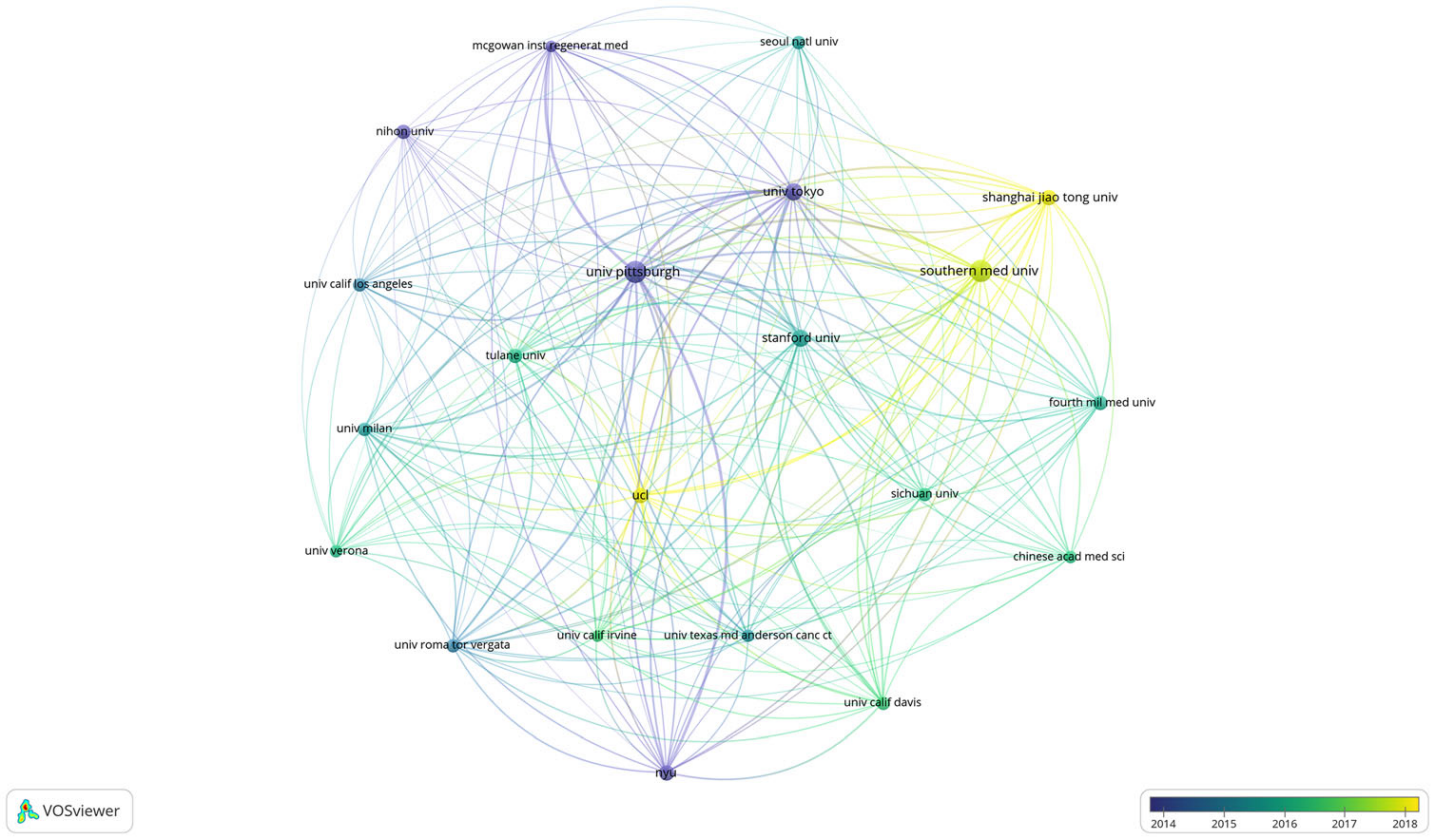
Chronological order of affiliation collaboration network.

To exhibit the cooperation among the different affiliations, the VOSviewer was used to make an overlay diagram and a density map as shown in Fig. 4 and Fig. S2. After setting the minimum number of documents for one affiliation as 10, 21 affiliations met the criteria. The affiliations published articles mainly in 2014 and the top 10 affiliations publishing the most documents were the center in the field of ASCs and fat grafting.

### Distribution of Journals

**Fig. 5:**
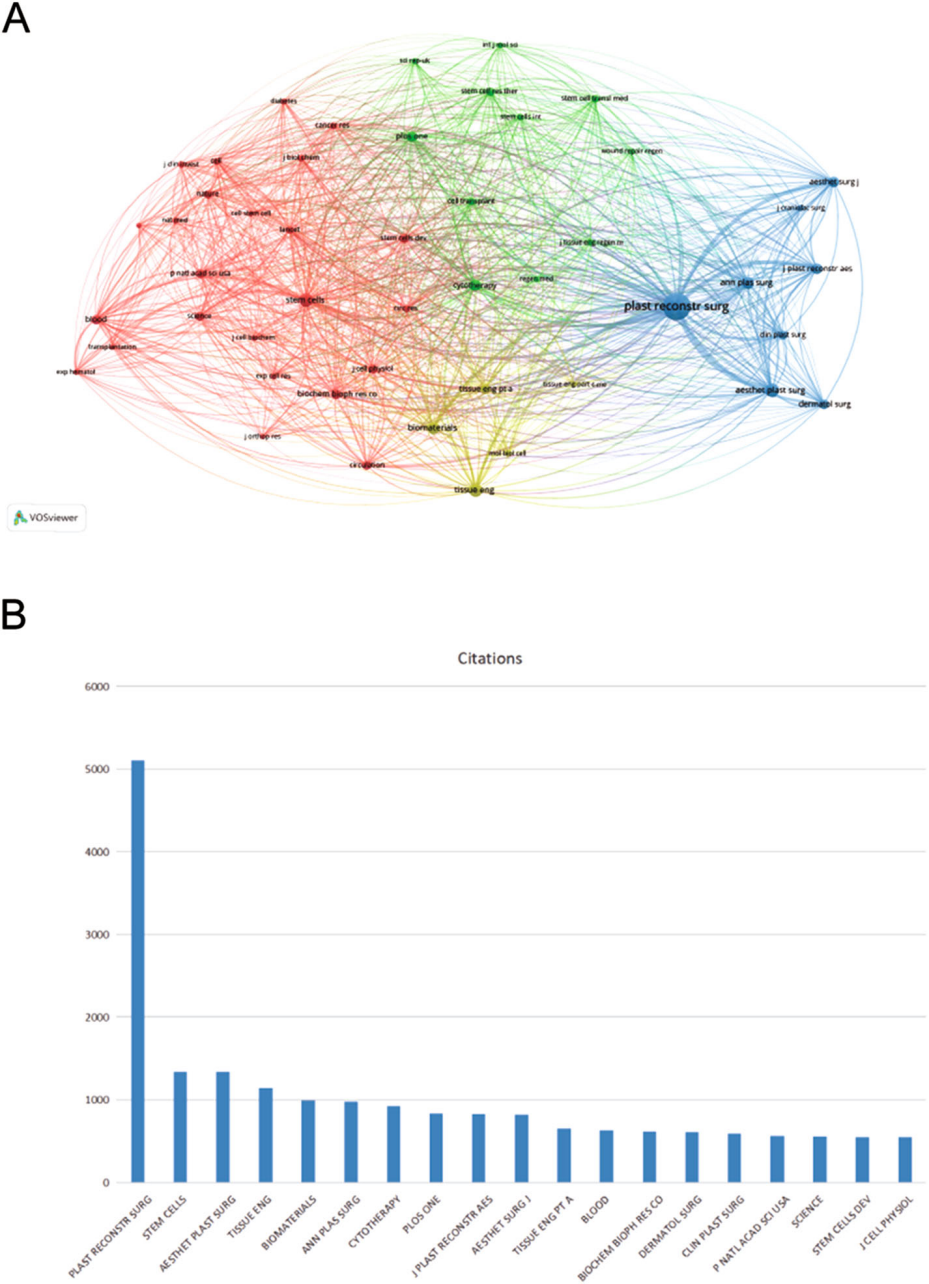
**A** Co-citation network visualization of references. **B** Top 20 most cited journals.

The top 10 journals publishing the most articles about ASCs and fat grafting are listed in Table 4. Plastic and Reconstructive Surgery (IF = 5.169) with 98 publications ranked first, followed by Aesthetic Surgery Journal (IF = 4.485) with 54 publications and Annals of Plastic Surgery (IF = 1.763) with 42 publications. About 30.96% documents were published in these top 10 journals and Plastic and Reconstructive Surgery had the highest H-index (H-index = 40). To directly show the complex relationships among the journals, co-citation analysis was performed using VOSviewer. Co-citation of journals indicated that two publications in different journals received a citation from a third publication published in another journal [5]. The minimum number of citations for a source was set as 200, and 48 journals were included in further analysis (Fig. 5A). The top 20 most cited journals are shown in Fig. 5B. Plastic and Reconstructive Surgery was the most cited journal with 5436 citations, followed by Aesthetic Plastic Surgery (IF = 2.7, 1441 citations) and Aesthetic Surgery Journal (IF = 4.48, 1205 citations).

**Table 4.** Top 10 journals according to publications.

Rank	Journal	Np	% of (1166)	Nc	H-index	IF (2021)
1	Plastic and Reconstructive Surgery	98	8.40%	5436	40	5.169
2	Aesthetic Surgery Journal	54	4.63%	1205	23	4.485
3	Annals of Plastic Surgery	42	3.60%	1097	17	1.763
4	Aesthetic Plastic Surgery	35	3.00%	1441	15	2.708
5	Stem cell Research Therapy	27	2.32%	818	13	8.079
6	Journal of Tissue Engineering and Regenerative Medicine	23	1.97%	488	13	4.323
7	Journal of Craniofacial Surgery	22	1.89%	501	10	1.172
8	Journal of Plastic Reconstructive and Aesthetic Surgery	22	1.89%	1008	13	3.022
9	Cell Transplantation	19	1.63%	768	15	4.139
10	Plos One	19	1.63%	618	13	3.752

**Fig. 6:**
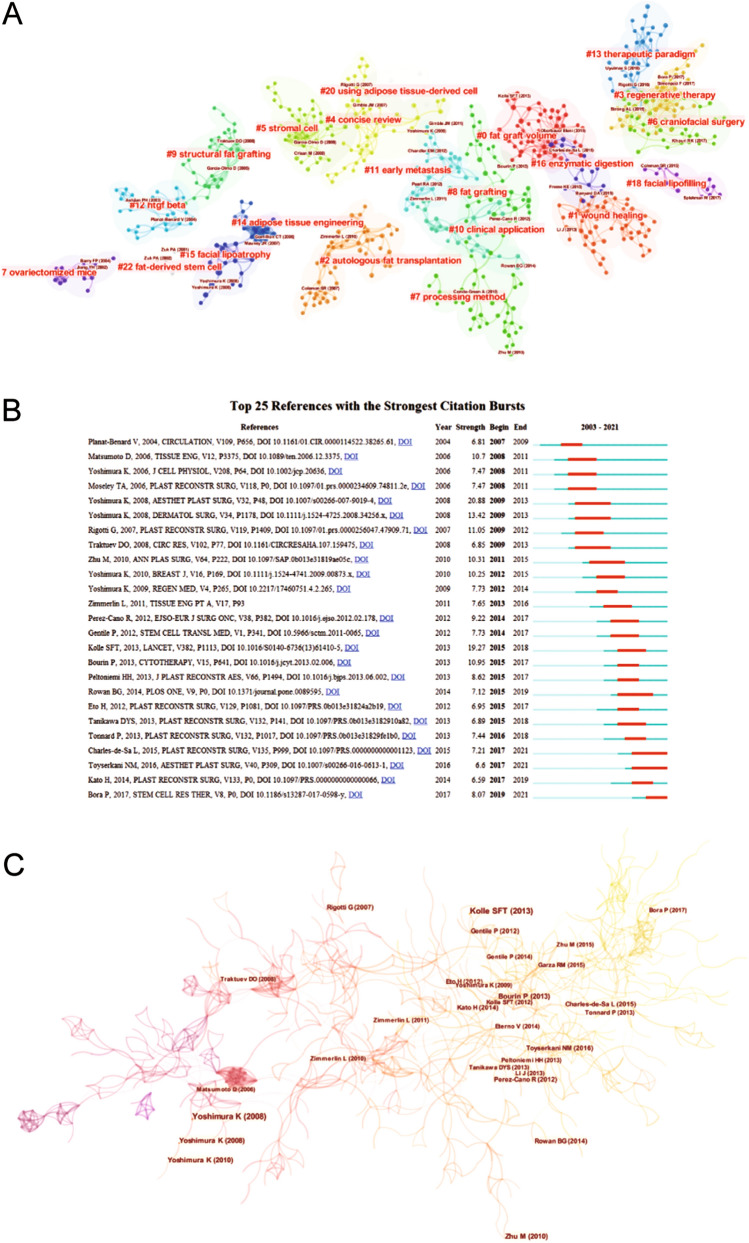
**A** A simplified co-citation network visualization of the references. **B** top 25 references with the most citation bursts calculation. **C** Co-citation network title clustering analysis.

Then we performed the co-citation references analysis using CiteSpace software and the network visualization is shown in Fig. 6A. The central node was the article “Enrichment of autologous fat grafts with ex vivo expanded adipose tissue-derived stem cells for graft survival: a randomised placebo-controlled trial” published by Stig-Frederik Trojahn Kølle [15]. The second centric node was “Cell-assisted lipotransfer for cosmetic breast augmentation: Supportive use of adipose-derived stem/stromal cells” published by Kotaro Yoshimura [4]. Both articles conducted a comprehensive and rigorous study on the efficiency and safety of ASCs in fat transplantation. We further used the burst detection function of CiteSpace software to observe the citation changes over time. The top 25 references with the strongest citation bursts are listed in Fig. 6B. We could see from the data that the number of citations was stable with no rapid change over the 20-year period. Then we conducted cluster analysis based on the titles of the references and the co-citation network is shown in Fig. 6C. It was clustered into several categories, including “fat graft volume,” “wound healing,” “autologous fat transplantation,” “regenerative therapy,” “concise review,” “stromal cell,” “craniofacial surgery,” “processing method,” “fat grafting,” “structural fat grafting,” “clinical application,” “early metastasis,” “htgf beta,” “therapeutic paradigm,” “adipose tissue engineering,” “facial lipoatrophy,” “enzymatic digestion,” “ovariectomized mice,” “facial lipofilling,” “using adipose-tissue-derived cells” and “fat-derived stem cells.” As shown in Fig. S3, “therapeutic paradigm,” “craniofacial surgery” and “regenerative therapy” have raised considerable concerns in recent years.

### Distribution of Authors and Their Collaborations

The top 10 most accomplished authors in the field of ASCs and fat grafting are listed in Table 5, most of which were from USA. They published 178 articles in total accounting for 15.27% of the whole publications. Rubin, J Peter ranked first with a notably high H-index and Nc (H-index = 20, Nc = 1291), followed by Lu Feng and Gentile Pietro.

**Table 5.** Top 10 authors according to publications.

Rank	Author	Country	Np	% of (1166)	Nc	H-index
1	Rubin, J Peter	USA	27	2.32%	1291	20
2	Lu, Feng	China	23	1.97%	361	12
3	Gentile, Pietro	Italy	21	1.80%	1210	18
4	Marra, Kacey	USA	21	1.80%	907	17
5	Longaker, Michael T.	USA	18	1.54%	818	14
6	Wan, Derrick C.	USA	14	1.20%	448	11
7	Gao, Jianhua	China	14	1.20%	360	9
8	Yoshimura, Kotaro	Japan	14	1.20%	1294	12
9	Gimble, Jeffrey M.	USA	14	1.20%	584	12
10	Kelly, Daniel J.	Ireland	12	1.03%	345	10

Based on the number of co-authored documents, the total link strength between the authors was calculated. Among the 5831 authors of the 1166 articles, 111 authors who published no less than 5 articles were included in further analysis (Fig. S4). Ranking by the total link strength, "Yoshimura Kotaro" (Total link strength = 91), "Kato Harunosuke" (Total link strength = 64) and "Longaker Michael" (Total link strength = 60) were in the top 3 positions.

### Distribution of paper local citation scores

Local citation score (LCS) was a key indicator of contribution, calculated based on the Nc of one publication in a specific area [16]. Higher LCS of a publication could partly indicated higher degree of innovation in the corresponding knowledge field [10]. The top 10 articles with the highest LCS are listed in Table 6. The article written by Yoshimura K in 2008 had the highest LCS of 35 in 2015 [4]. In this article, the authors found that the cell-assisted lipotransfer is safe and efficient for soft tissue augmentation and better than traditional lipoinjection.

**Table 6:** Top 10 references according to LCS.

Rank	Title	Author	Journal	Year	LCS
1	Cell-assisted lipotransfer for cosmetic breast augmentation: Supportive use of adipose-derived stem/stromal cells	Yoshimura, K	AESTHETIC PLASTIC SURGERY	2008	261
2	Cell-assisted lipotransfer: Supportive use of human adipose-derived cells for soft tissue augmentation with lipoinjection	Matsumoto, D	TISSUE ENGINEERING	2006	216
3	Cell-assisted lipotransfer for facial lipoatrophy: Efficacy of clinical use of adipose-derived stem cells	Yoshimura, K	DERMATOLOGIC SURGERY	2008	154
4	Enrichment of autologous fat grafts with ex-vivo expanded adipose tissue-derived stem cells for graft survival: a randomised placebo-controlled trial	Kolle, SFT	LANCET	2013	147
5	Structural fat grafting: More than a permanent filler	Coleman,SR	PLASTIC AND RECONSTRUCTIVE SURGERY	2006	144
6	The Fate of Adipocytes after Nonvascularized Fat Grafting: Evidence of Early Death and Replacement of Adipocytes	Eto, H	PLASTIC AND RECONSTRUCTIVE SURGERY.	2012	122
7	Supplementation of Fat Grafts With Adipose-Derived Regenerative Cells Improves Long-Term Graft Retention	Zhu, M	ANNALS OF PLASTIC SURGERY	2010	105
8	Adipose-derived stem and progenitor cells as fillers in plastic and reconstructive surgery	Moseley TA	PLASTIC AND RECONSTRUCTIVE SURGERY.	2006	84
9	Progenitor-Enriched Adipose Tissue Transplantation as Rescue for Breast Implant Complications	Yoshimura, K	BREAST JOURNAL	2010	73
10	Nanofat Grafting: Basic Research and Clinical Applications	Tonnard P	PLASTIC AND RECONSTRUCTIVE SURGERY.	2013	72

### Distribution of Research Hot spots

**Fig. 7:**
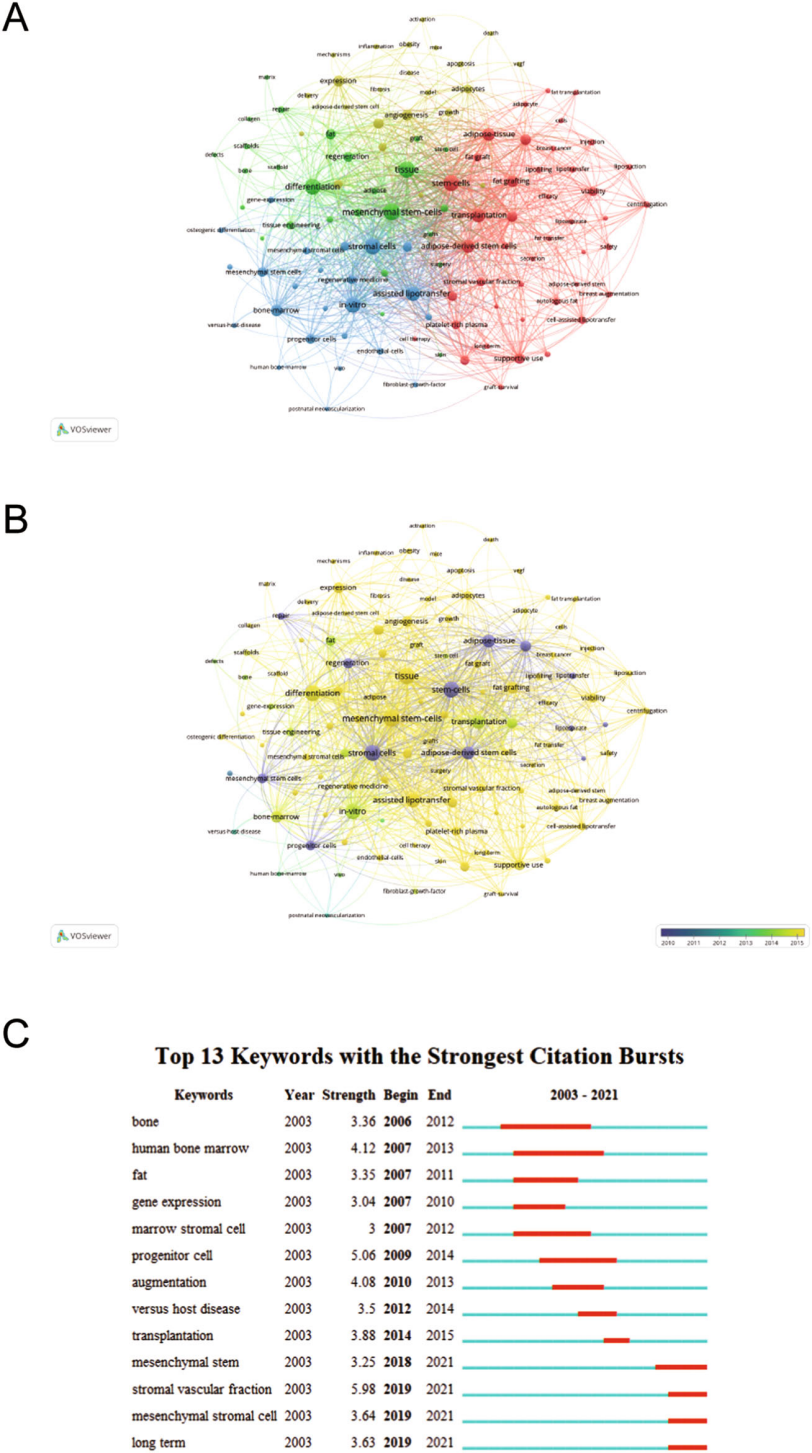
**A** Co-occurrence network of keywords in of adipose-derived stem cells in fat grafting study. **B** Chronological order of keyword co-occurrence network. **C** Top 13 keywords with the most citation bursts calculation.

To have a systematic recognition of the research hot spots and developmental direction of ASCs and fat grafting, we conducted the co-occurrence cluster analysis based on the keywords using the VOSviewer software. After filtered by the threshold of 20, a total of 116 keywords were included in further visual analysis. A network map was made and is presented in Fig. 7A. Cluster 1 (Red) was mainly related to adipose-tissue grafting. Cluster 2 (Green) was primarily about mesenchymal stem cell. Cluster 3 (Blue) was mainly concerning stromal cell-assisted lipotransfer. Cluster 4 (Yellow) was principally regarding cellular state of ASCs. Moreover, Fig. 7B indicates that “mesenchymal stem-cell,” “stem-cells,” “adipose-derived stem cells” and “transplantation” were the interesting hot spots in the field of ASCs and fat grafting recently. In addition, as presented in Fig. S5, “breast augmentation,” “growth,” “versus host disease,” “differentiation,” “fat,” “vascularization,” “osteoblast” and “engraftment” were the most popular keywords for a long time of the studies in this field. Then as shown in Fig. 7C, “mesenchymal stem,” “mesenchymal stromal cell,” “stromal vascular fraction” and “long term” were the most trending keywords in recent time.

## Discussion

According to the literature data retrieved from the WoSCC database, we conducted bibiometric analyses and presented the visualization results of the research status and trend about ASCs and fat grafting. A total of 1166 articles in English were searched out from 2002-01-01 to 2021-12-31. A number of articles were published annually, and their citations kept increasing overtime. It demonstrated that researchers were of great interests in ASCs and fat grafting which was a prospective research hot spot. There were 30 countries/regions that have published no less than 5 articles in this field, among which USA ranked first according to Np, Nc and H-index. Most of the affiliations and authors ranking by the front based on the number of publications were from USA, indicating that USA took the lead of the research in this area. University of California System (Np = 49, Nc = 2014), Pennsylvania Commonwealth System of Higher Education PCSHE (Np = 37, Nc = 2182) and Southern Medical University China (Np = 37, Nc = 654) were the leading institutions. Rubin Peter, Lu Feng and Gentile Pietro published most articles, of which the article entitled “Enrichment of autologous fat grafts with ex vivo expanded adipose tissue-derived stem cells for graft survival: a randomised placebo-controlled trial” published by Stig-Frederik Trojahn Kølle from Copenhagen University Hospital pointed out an important discovery in this field. The research indicated that the procedure of adipose-derived stem cells enriched fat transplantation is feasible and safe. Enrolled 13 participants. In comparision with control group, the ASCs enriched fat transplantation had remarkable higher residual volumes of fat [15].

Up to half of the top 10 productive journals had Impact factor (IF) more than 5, demonstrating that the research outputs were of high quality. Plastic and Reconstructive Surgery, Aesthetic Surgery Journal and Annals of Plastic Surgery published the largest number of related articles. Plastic and Reconstructive Surgery was a classical journal providing up-to-the-minute knowledge about the latest techniques and significant developments for all aspects of plastic and reconstructive surgery, such as breast reconstruction, maxillofacial reconstruction and burn repair. From this journal, the article entitled “Structural fat grafting: More than a permanent filler” had as high as 760 citations [17]. In this research, authors have indicated that stem cells can repair and even become cartilage, blood vessels, muscles, bones, nerves and skin. Further research is essential for understanding the transplanted adipose tissue. Aesthetic Surgery Journal was cited 1205 times, from which the article entitled “Effects of centrifugation on cell composition and viability of aspirated adipose tissue processed for transplantation” was mostly cited [18]. This article discussed the centrifugation method in fat processing and its influence on the reservation of mesenchymal stem cells which played a key role in angiogenic and adipogenic effect of the grafted adipose tissue [18].

Among the top 10 articles with the highest LCS, half of them were published in high-IF journals. The article entitled “Cell-assisted lipotransfer for cosmetic breast augmentation: supportive use of adipose-derived stem/stromal cells” written by Yoshimura K had the highest LCS (LCS = 261) [4]. In this study, researchers brought autologous ASCs in lipoinjection treatment and demonstrated its efficacy and safety for soft tissue augmentation [4]. In 2013, the result of a randomized controlled trial was published in Lancet named “Enrichment of autologous fat grafts with ex vivo expanded adipose tissue-derived stem cells for graft survival: a randomized placebo-controlled trial” (LCS = 147) [15]. This trial indicated that fat grafts enriched with ASCs had significantly higher residual volume after injected and the method was relatively feasible and safe [15]. These publications provided some promising directions which deserved further in-depth studies.

The popular objectives around ASCs and fat grafting were evaluated by cluster analyses of the keywords of correlational studies. Our results showed that adipose-derived stem cells, mesenchymal stem cells and transplantation were the hot spots in this area.

In general, the bibliometric analysis summarized the temporal, regional and quantitative characteristics of the published articles in the area of ASCs and fat grafting. Also, it presented the hot spots of current studies and pointed out further development perspectives in this research field. However, this study still had some limitations. First, our study only included publications in English with the document type of article and review from SCI-expanded WoSCC database. The exclusion of other document types might omit some data. Second, limited by the VOSviewer and CiteSpace software, we could not analyze the complete content of the articles, so the information might be half-baked. Third, the citation number influenced by many factors could not equal to research quality. At last, as publication was a time-consuming process, so the bibiometric analyses could not offer the most up-to-date results. Some good quality articles which have just been published might be ignored due to their low Nc. So the bibiometric summary should be updated periodically.

## Supplementary Information

Below is the link to the electronic supplementary material.

Supplementary file1 (DOCX 3552 KB)

## Data Availability

This study used data from the Web of Science Core Collection database.
