# Supplementary material for: Global Analyses and Latest Research Hot Spots of Adipose-Derived Stem Cells in Fat Grafting: A Bibliometric and Visualized Review
Source: Aesthetic Plast Surg. 2022 Dec 2;47(3):1192–204. doi: 10.1007/s00266-022-03201-1 (PMC10229469; doi:10.1007/s00266-022-03201-1)
Supplement: Supplementary file 1 — Supplementary file1 (DOCX 3552 KB) [file 266_2022_3201_MOESM1_ESM.docx]

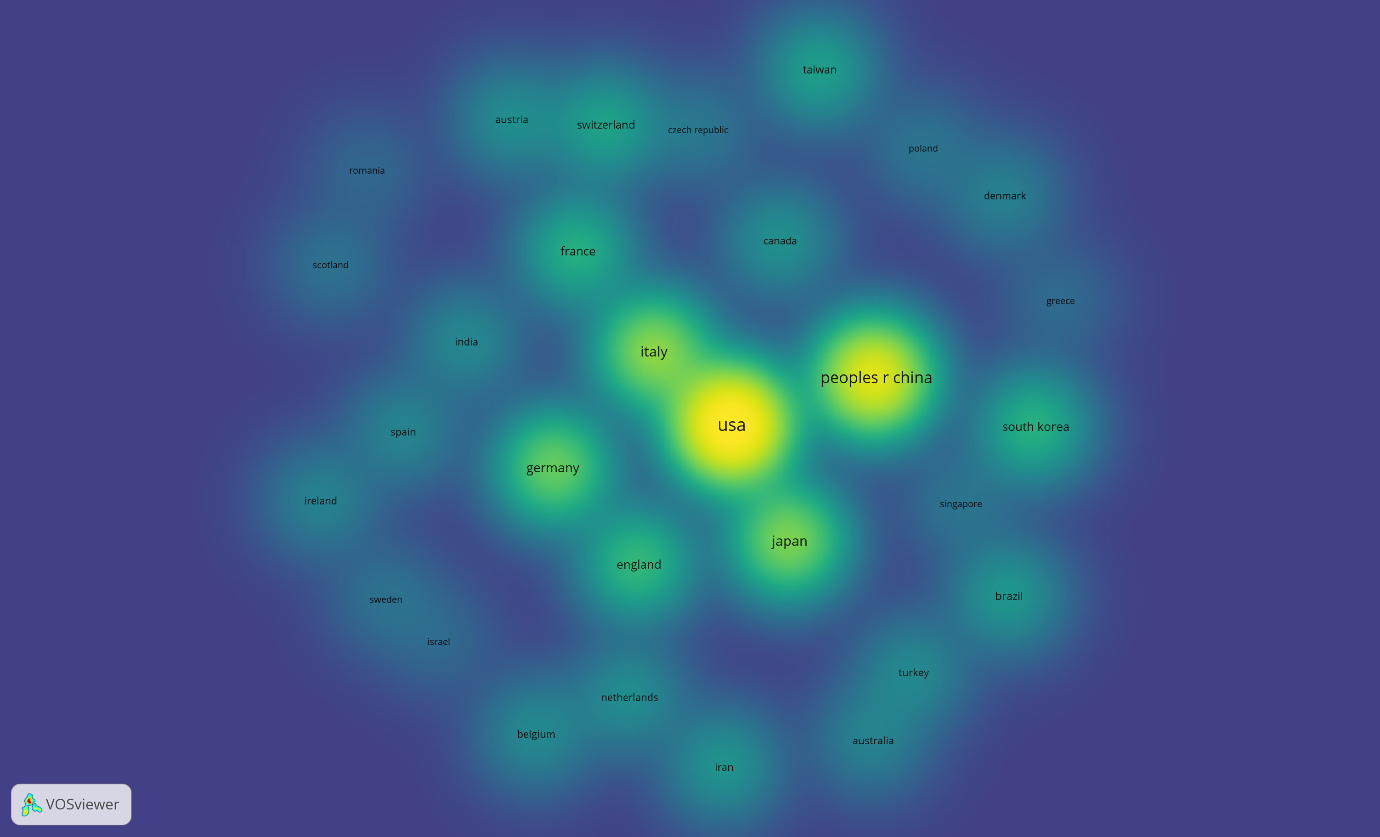


Figure S1. Density visualization for global country distribution analysis.


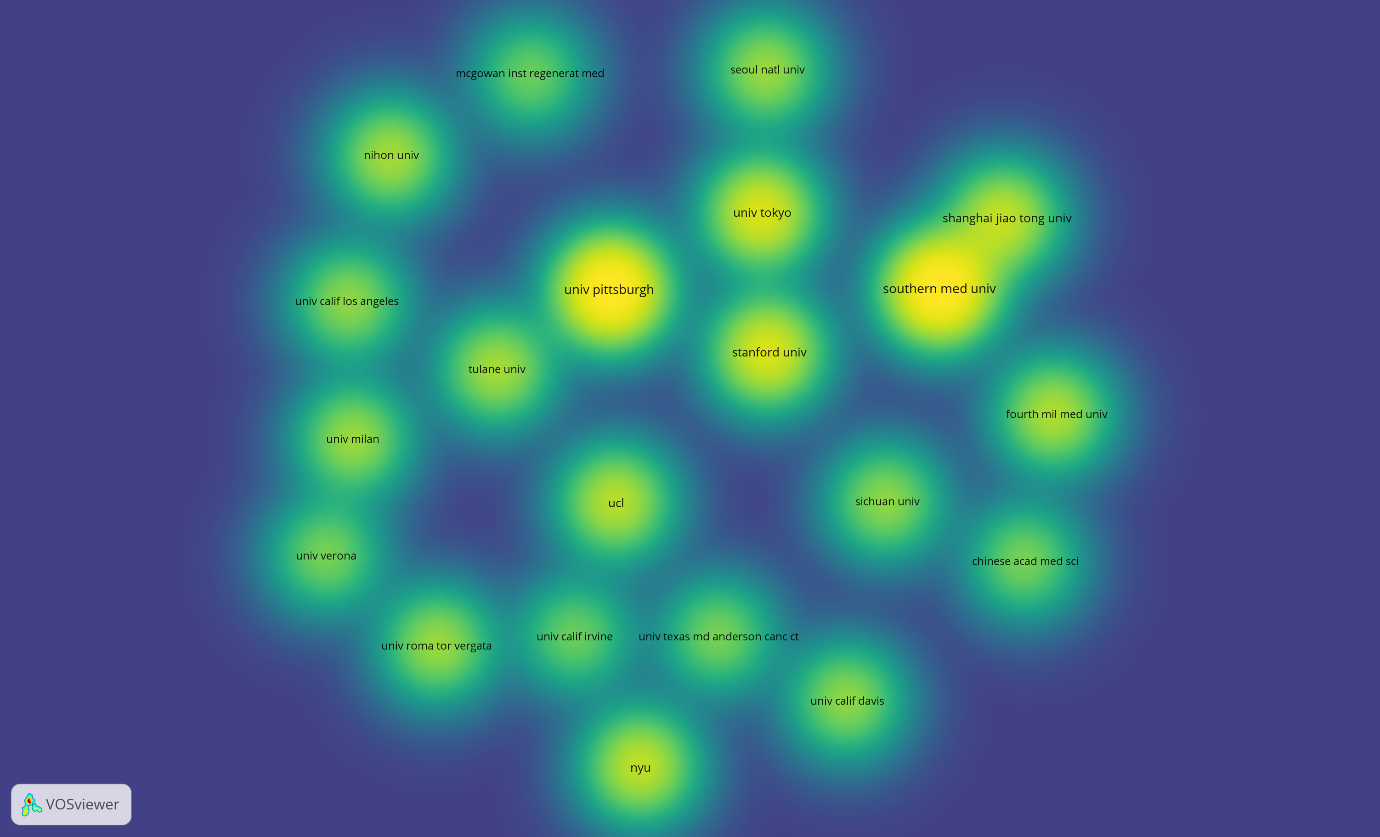


Figure S2. Density visualization for affiliation collaboration analysis.


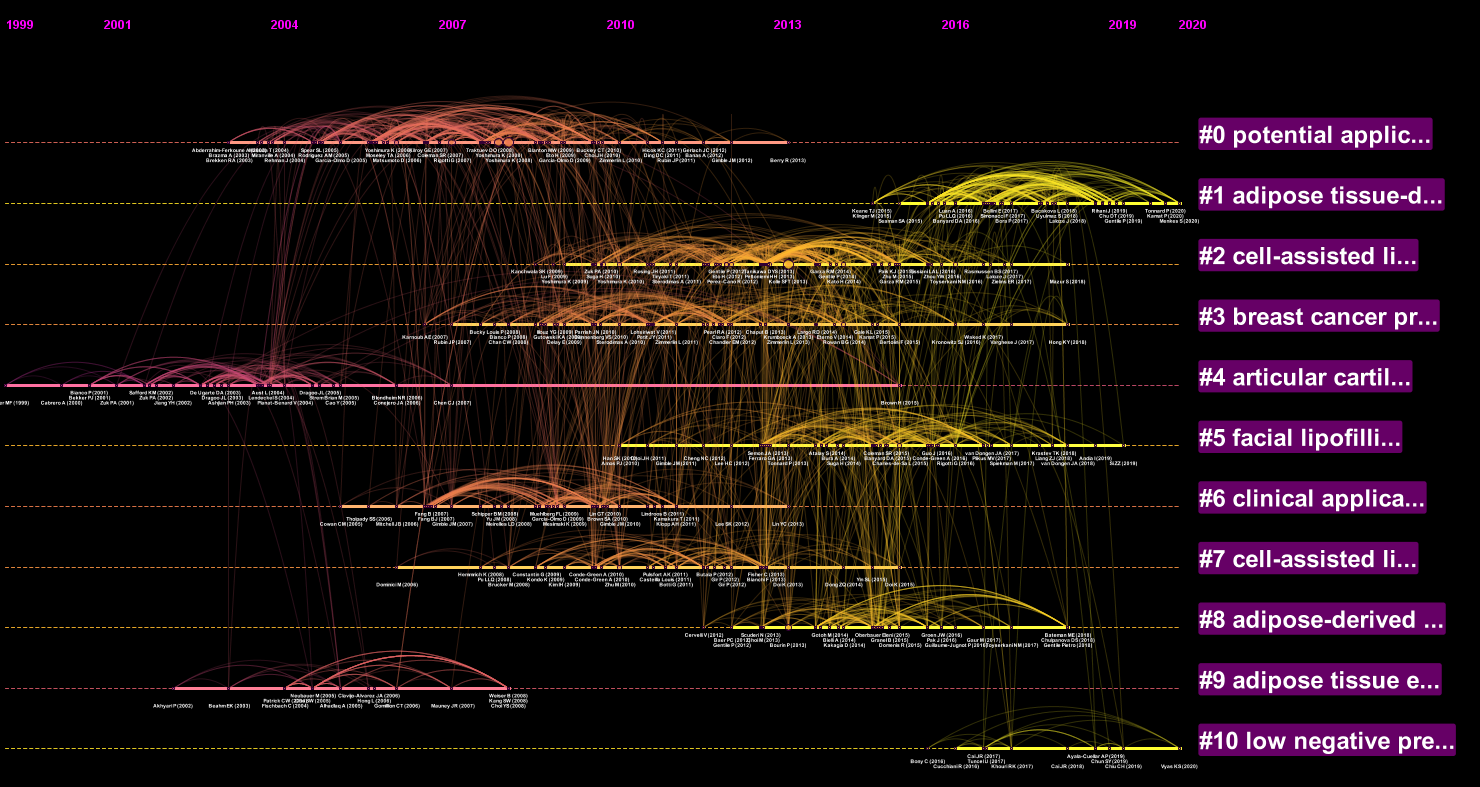


Figure S3. Timeline visualization of references title clustering from 2002 to 2021.


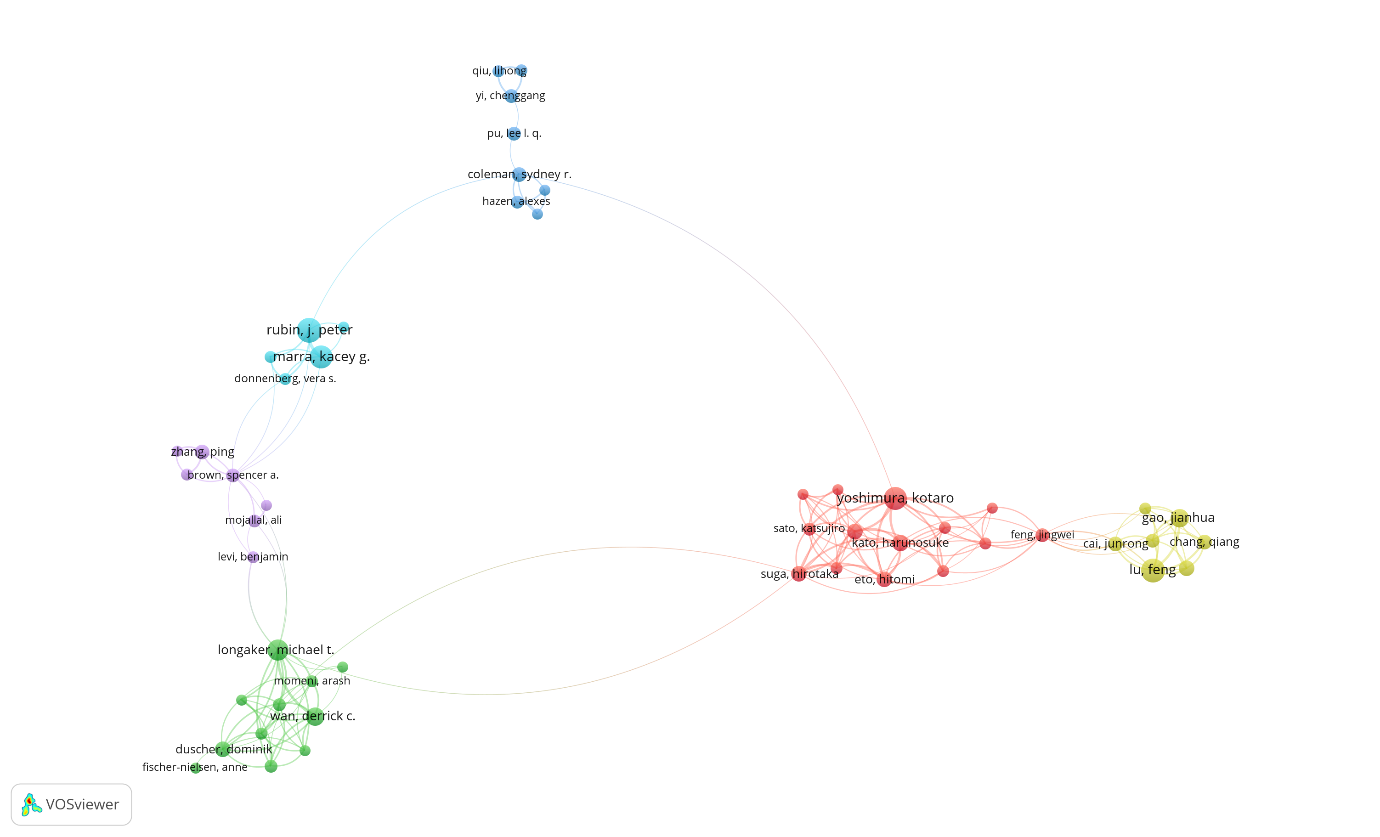


Figure S4. Cluster visualization map of Co-authorship in research of of adipose-derived stem cells in fat grafting from 2002 to 2021.


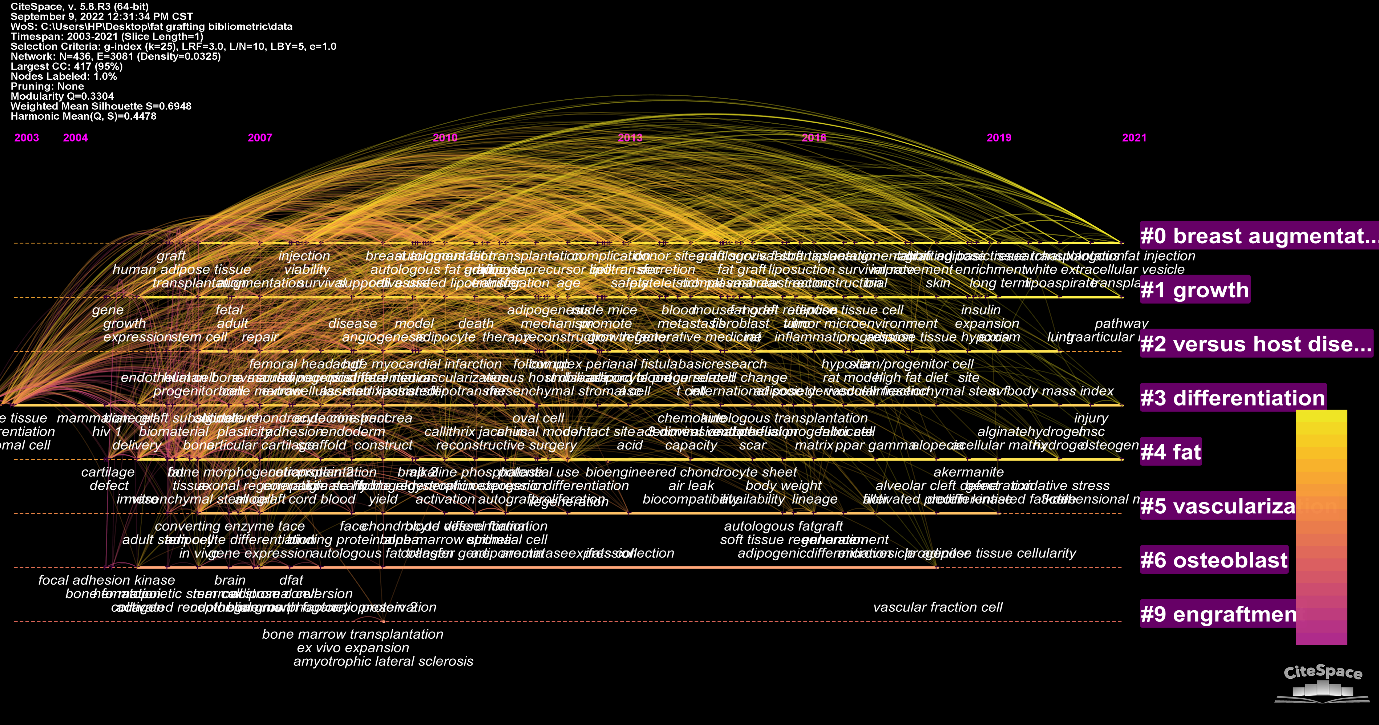


Figure S5. Timeline visualization of keywords clustering from 2002 to 2021.
